# Chimeric 3’ flanking regions strongly enhance gene expression in plants

**DOI:** 10.1111/pbi.12931

**Published:** 2018-05-21

**Authors:** Andrew G. Diamos, Hugh S. Mason

**Affiliations:** ^1^ Center for Immunotherapy, Vaccines and Virotherapy Biodesign Institute at ASU, and School of Life Sciences Arizona State University Tempe AZ USA

**Keywords:** 3’ UTR, biopharmaceutical, geminiviral vector, gene terminator, matrix attachment region, recombinant protein, transient expression

## Abstract

Plants represent a promising platform for the highly scalable production of recombinant proteins. Previously, we identified the tobacco extensin terminator lacking its intron as an element that reduced transcript read‐through and improved recombinant protein production in a plant‐based system. In this study, we systematically compared nonreplicating plant expression vectors containing over 20 commonly used or newly identified terminators from diverse sources. We found that eight gene terminators enhance reporter gene expression significantly more than the commonly used 35S and NOS terminators. The intronless extensin terminator provided a 13.6‐fold increase compared with the NOS terminator. Combining terminators in tandem produced large synergistic effects, with many combinations providing a >25‐fold increase in expression. Addition of the tobacco Rb7 or TM6 matrix attachment region (MAR) strongly enhanced protein production when added to most terminators, with the Rb7 MAR providing the greatest enhancement. Using deletion analysis, the full activity of the 1193 bp Rb7 MAR was found to require only a 463‐bp region at its 3’ end. Combined terminators and MAR together provided a >60‐fold increase compared with the NOS terminator alone. These combinations were then placed in a replicating geminiviral vector, providing a total of >150‐fold enhancement over the original NOS vector, corresponding to an estimated yield of 3–5 g recombinant protein per kg leaf fresh weight or around 50% of the leaf total soluble protein. These results demonstrate the importance of 3’ flanking regions in optimizing gene expression and show great potential for 3’ flanking regions to improve DNA‐based recombinant protein production systems.

## Introduction

Plant‐based recombinant protein production systems have emerged as promising alternatives to traditional mammalian and microbial cell culture systems due to unique advantages of lower costs, high scalability and improved safety (Chen and Davis, 2016; Komarova *et al*., 2010). Case studies have shown the potential for large cost reductions in capital investment and the cost of goods for plant‐made therapeutics compared with conventional methods (Nandi *et al*., 2016; Tusé *et al*., 2014). The capacity for these systems to rapidly and safely produce therapeutics has been demonstrated by two success stories: the FDA approval of an enzyme replacement therapy for Gaucher's disease, which became the first plant‐made therapeutic (Fox, 2012; Zimran *et al*., 2011); and the monoclonal antibody therapy ZMapp given during the 2014 Ebola outbreak, which was shown to protect against lethal virus challenge (Lyon *et al*., 2014; Qiu *et al*., 2014). Many strategies for improving protein production in plants have been explored, such as viral expression systems, subcellular targeting, agrobacterium strain, expression host, promoters, introns and 5’ untranslated regions (UTR). However, another key component in many of these systems is the gene terminator and surrounding regions, which have not been systematically optimized.

The gene terminator determines the fate of each transcript through a complex interplay of nuclear and cytoplasmic processes. Polyadenylation is a well‐studied process involving cleavage of the nascent mRNA strand and the addition of a tract of 100–200 adenosine residues, forming the poly(A) tail. Plant polyadenylation requires the coordinated action of three main genetic signals: the UA‐rich cleavage element, which contains the cleavage and polyadenylation site; an A‐rich near upstream element; and a UG‐rich far upstream element. Alteration of any of these three signals can drastically impact gene expression (Hunt, 2008; Loke, 2005; Mathew *et al*., 2011; Rothnie, 1996). Polyadenylation also plays a pivotal role in transcription and translation. The 3’ end of the mRNA and accompanying processing machinery are in close contact with the 5’ end of the gene, a phenomenon known as gene looping. By this mechanism, efficient polyadenylation has been linked to enhanced transcription through RNA Pol II recycling (Andersen *et al*., 2012; Mapendano *et al*., 2010; Tan‐Wong *et al*., 2009, 2012). Quality control of mRNA is also highly dependent on 3’‐end processing, as improperly polyadenylated transcripts are targeted for nuclear degradation. Furthermore, the 3’‐end processing machinery participates in coordinating the assembly of an export‐competent mRNP (Libri *et al*., 2002; Moore and Proudfoot, 2009; Qu *et al*., 2009; Singh *et al*., 2015). Poly(A) binding proteins, along with numerous other proteins which interact with the 3’ UTR, play an essential role in translation by participating in translation initiation factor recruitment, ribosome recycling and mRNA decay (Wigington *et al*., 2014).

The terminator can profoundly affect gene silencing. RNA‐dependent RNA polymerase 6 (RDR6) plays a key role in synthesizing dsRNA from overexpressed viral or transgene transcripts to initiate or amplify the silencing cascade leading to transcript degradation (Allen and Howell, 2010; Qin *et al*., 2012). Improperly processed transcripts producing high levels of read‐through transcription were targeted for silencing by this pathway (Luo and Chen, 2007). Additionally, a study that combined two terminators in tandem found a large increase in protein production compared with either terminator alone, possibly as a result of reduced read‐through transcription leading to reduced gene silencing (Beyene *et al*., 2011). RDR6 also has affinity for transcripts lacking proper polyadenylation, further underscoring the importance of the 3’ UTR in mitigating gene silencing (Baeg *et al*., 2017).

Besides the gene terminator, other 3’ genetic elements contribute to gene expression in plants. Chromatin scaffold/matrix attachment regions (MAR) are AT‐rich regions that associate with the nuclear matrix and are thought to affect higher‐order chromatin structure (Calikowski *et al*., 2003; Halweg *et al*., 2005; Liebich *et al*., 2002). The tobacco Rb7 MAR, when placed at both the 5’ and 3’ ends of the expression cassette, increased the likelihood and magnitude of GFP production in tobacco cells (Halweg, 2005). Additionally, MAR have been implicated in the reduction of transgene silencing (Mlynárová *et al*., 2003). The tobacco TM6 MAR reduced repressive DNA methylation in flanking promoter regions and enhanced recombinant protein production in transgenic tobacco (Ji *et al*., 2013). We found that the Rb7 MAR reduced leaf cell death and substantially increased protein production in a replicating geminiviral transient expression system, but the effect required the MAR to be positioned at the 3’ end of the gene terminator (Diamos *et al*., 2016). The potential of MAR to enhance protein production in transient expression systems has not been thoroughly investigated.

Despite the well‐established critical role of gene terminators in facilitating high levels of gene expression, few studies have focused on the gene terminator in the context of recombinant protein production. For nearly 30 years, the most widely used terminators have been the nopaline synthase (NOS) and octopine synthase (OCS) terminators from *Agrobacterium tumefaciens*, and the 35S terminator from cauliflower mosaic virus (MacFarlane *et al*., 1992; Ellis *et al.,* 1987; Pietrzak *et al*., 1986). In transgenic potato plants, the 3’ UTR from the potato *pinII* gene provided 10–50 times greater accumulation of hepatitis B surface antigen compared with the NOS terminator (Richter *et al*., 2000). More recently, the terminator from a heat‐shock protein from *Arabidopsis* (AtHSP) was found to provide a twofold increase in GUS production in transient or stable transgenic *Arabidopsis* plants compared with NOS (Nagaya *et al*., 2010). Previously, we identified the tobacco extensin terminator, which reduced transcript read‐through and improved transient expression substantially compared with the NOS or 35S terminators, an effect which was amplified when its native intron was removed Rosenthal *et al*., (2010). In this study, we systematically investigate the potential of the gene terminator and 3’ flanking region to improve plant‐based expression comparing expression vectors containing diverse terminators from plant and viral sources.

## Results

### Evaluation of diverse terminators on GFP production

**Figure 1:**
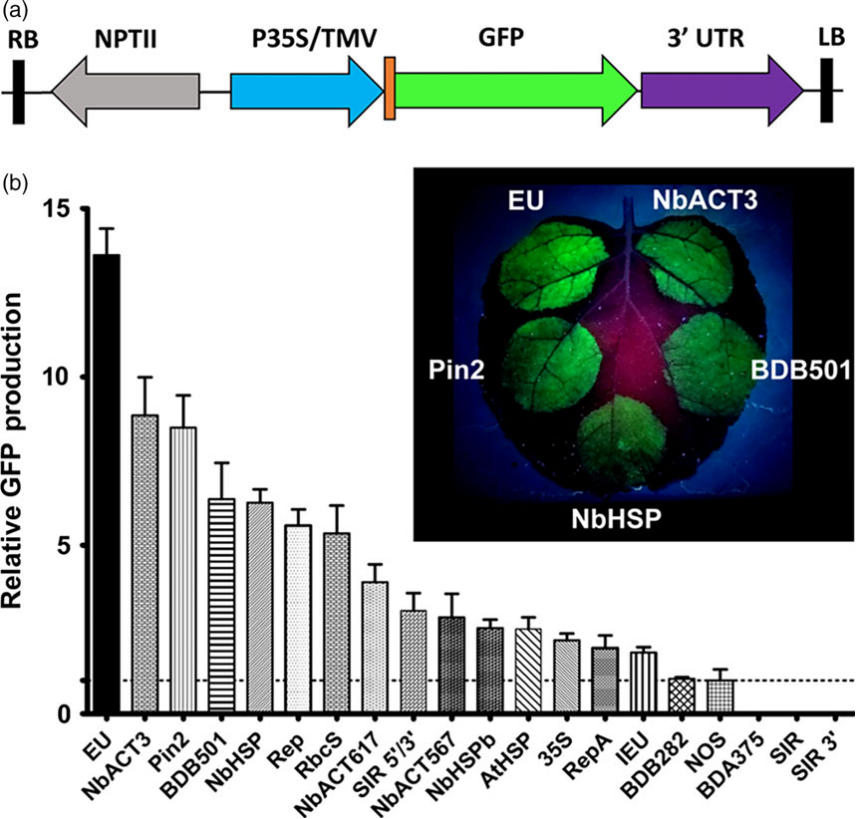
Evaluation of diverse 3’ UTRs on GFP production. (a) Generalized schematic representation of the T‐DNA region of the vectors used in this study. RB and LB, the right and left borders of the T‐DNA region; NPTII, kanamycin resistance cassette; P35S, 35S promoter from cauliflower mosaic virus; TMV, 5’ UTR from tobacco mosaic virus; 3’ UTR is either a single terminator, double terminator, matrix attachment region or combination of these elements as described in each experiment. (b) Nonreplicating vectors containing 3’ UTRs inserted downstream from the GFP gene were agroinfiltrated into *N. benthamiana* leaves. Leaves were photographed at 5 DPI under UV illumination (365 nm). Images are representative of 3–4 independently infiltrated leaves. Agroinfiltrated leaves were harvested between 4 and 5 DPI and extracts were analysed by SDS‐PAGE followed by observation under UV illumination (365 nm) and Coomassie staining. GFP band intensity was quantified using ImageJ software, using native plant protein bands as a loading control. Data are means ± SE of 3–4 independently infiltrated leaves. Abbreviations: EU, intronless tobacco extensin 3’ UTR; IEU, intron‐containing tobacco extensin 3’ UTR; NbACT3, *N. benthamiana* actin 3’ UTR; NbHSP;* N. benthamiana* 18.8 kDa class II heat‐shock protein 3’ UTR; pinII, potato proteinase inhibitor II 3’ UTR; rbcS, pea rubisco small subunit 3’ UTR; SIR, short intergenic region of bean yellow dwarf virus; BDB, bean dwarf mosaic virus DNA B nuclear shuttle protein 3’ UTR; Rep, bean dwarf mosaic virus rep gene 3’ UTR; RepA, bean dwarf mosaic virus repA gene 3’ UTR; AtHSP,* A. thaliana* heat‐shock protein 3’ UTR; 35S, cauliflower mosaic virus 35S 3’ UTR; NOS, agrobacterium nopaline synthase 3’ UTR.

To systematically evaluate diverse terminators, we constructed expression vectors using 20 different terminators from plant and viral sources, placed 3’ of a GFP reporter gene, which was driven by the strong 35S promoter and tobacco mosaic virus 5’ UTR (Figure 1a). These constructs were delivered to *N. benthamiana* leaves by agroinfiltration and evaluated for GFP production. To minimize leaf‐to‐leaf variation, each leaf was also infiltrated with a GFP vector containing the intronless tobacco extensin terminator (EU) as an internal control, which we previously found to be a potent enhancer of gene expression (Diamos *et al*., 2016; Rosenthal *et al*., 2018).

While much previous work used the NOS and 35S terminators, the *A. thaliana* 18.2 kDa heat‐shock protein terminator (AtHSP) was reported to enhance transgene production compared with the NOS terminator (Nagaya *et al*., 2010). In agreement with these results, we found that the AtHSP terminator provided a 2.5‐fold increase in GFP production compared with the NOS terminator (Figure 1b). Also consistent with previous work in transgenic potato (Richter *et al*., 2000), the potato *pinII* 3’ UTR provided a very strong 8.5‐fold increase compared with the NOS terminator (Figure 1b). The rubisco small subunit (rbcS) 3’ UTR from pea showed a 5.4‐fold enhancement. These data demonstrate that many terminators from diverse plant species have high activity in *N. benthamiana*.

To identify new candidates, a genomewide study of mRNA stability levels in *A. thaliana* (Narsai *et al*., 2007) was used to locate genes with potentially stability‐enhancing 3’ UTRs. We identified a *N. benthamiana* homolog of the *A. thaliana* 17.6 kDa class II heat‐shock protein (At5g12020). The 3’ flanking region from this homolog (referred to as NbHSP) was highly active, increasing reporter gene expression by 6.3‐fold compared with the NOS terminator, more than doubling the enhancement provided by the AtHSP terminator (Figure 1b). We also identified an *N. benthamiana* homolog of *A. thaliana* actin 7 (At5g09810), referred to as NbACT3. While the downstream 617‐nt region of NbACT3 enhanced expression by 3.9‐fold compared with NOS, extending the 3’ UTR to include more downstream sequence (1044 nt) resulted in a large 8.9‐fold enhancement (NbACT3, Figure 1b).

Many of the most highly active genetic elements in recombinant protein production systems are derived from viral sources. Therefore, we investigated the potential of viral terminators to enhance gene expression. The downstream short intergenic region (SIR) from the coat protein gene of bean yellow dwarf virus (BeYDV) showed no intrinsic terminator function by itself, or when additional downstream viral sequence (SIR 3’) was included (Figure 1b). However, when an upstream region (SIR 5’/3’) from the bean yellow dwarf virus coat protein coding sequence was also included, it was highly functional, providing GFP production threefold greater than the NOS terminator (Figure 1b). These data suggest that upstream elements present in the BeYDV coat protein gene are required for proper 3’ end processing. The downstream sequences from the BeYDV rep and repA genes were also found to be highly active, providing a 5.6‐fold and twofold respective enhancement. To test sequences from other geminiviruses, the terminators of bean dwarf mosaic virus (BDMV) genes were also investigated. A 282 nt sequence including the 3’ end of the nuclear shuttle protein, the intergenic region, as well as the 3’ end of the movement protein (BDB282) performed similarly to the NOS terminator. However, when an additional 200nt of the downstream movement protein sequence was included (BDB501), it provided a 6.4‐fold enhancement. A construct containing the BDMV coat protein downstream sequence alone was not functional, again suggesting that necessary signals may also reside in the gene coding sequence upstream from the terminator.

Taken together, these results show that many 3’ UTRs from diverse sources exceed the enhancement provided by the commonly used NOS or 35S terminators, at least in a transient expression system in *N. benthamiana* leaves. Consistent with our previous work, the EU terminator outperformed the other 19 3’ UTRs tested, providing a 13.6‐fold increase compared with the NOS terminator, indicating that it is a uniquely potent enhancer of gene expression.

### Combined gene terminators strongly enhance GFP production

**Figure 2:**
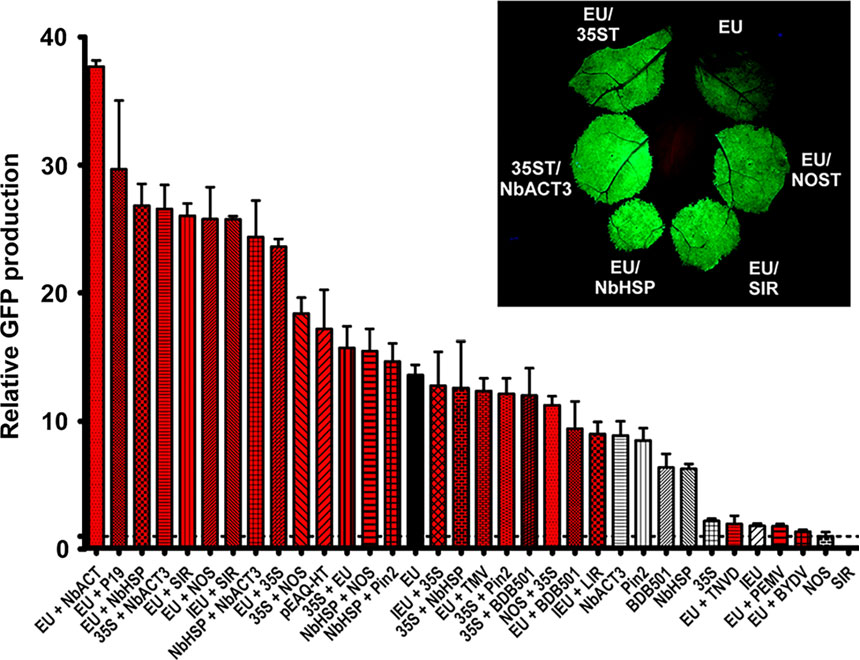
Double terminators strongly enhance GFP gene expression. Nonreplicating vectors containing different double terminators downstream from the GFP gene were agroinfiltrated into *N. benthamiana* leaves and analysed for GFP production at 5 DPI. Red bars indicate double terminators. Data are means ± SE of 3–4 independently infiltrated leaves. Abbreviations: BYDV, barley yellow dwarf virus 3’ UTR; PEMV, pea enation mosaic virus 3’ UTR; TNVD, tobacco necrosis virus‐D 3’ UTR; TMV, tobacco mosaic virus 3’ UTR; LIR, long intergenic region from bean yellow dwarf virus. For all other abbreviations, see Figure 1 legend.

A double terminator consisting of the 35S terminator fused to the NOS terminator greatly enhanced protein production in various plant species compared with either terminator alone (Beyene *et al*., 2011). To investigate the potential for tandem terminators to synergistically enhance recombinant protein production, we tested combinations of those previously tested in Figure 1b. We found an 18.4‐fold enhancement by the 35S‐NOS double terminator compared with the NOS terminator alone (Figure 2), exceeding the highest production by the best single terminator. Interestingly, reversing the position of the two terminators (NOS‐35S) provided a much lower 11.2‐fold enhancement, but still greatly exceeded the GFP production obtained with either terminator alone.

Fusion of 35S with pinII, NbHSP and BDB501 3’ regions all substantially enhanced protein production compared with either terminator alone. However, despite the individual superiority of each of these terminators compare to NOS, when paired with 35S, none exceeded the GFP production of 35S‐NOS (Figure 2, compare 35S‐Pin2, 35S‐NbHSP, 35S‐BDB501 and 35S‐NOS). In contrast, fusion of the individually strong NbACT3 terminator to the 3’ end of either the 35S or NbHSP terminators resulted in a potent enhancement of GFP production, exceeding the production of 35S‐NOS (Figure 2).

As the extensin EU terminator was the best individual terminator identified, we evaluated its potential combined with other terminators. Addition of either the NbHSP, NOS or 35S terminators to the 3’ end of the EU terminator nearly doubled the GFP production provided by EU alone, exceeding the gene expression provided by 35S‐NOS. The two best individual terminators, EU and NbACT3, when combined, exceeded all other combinations, providing a remarkable 37.7‐fold increase compared with NOS alone (Figure 2, EU + NbACT3). Interestingly, although the 35S terminator performed best when placed 5’ of the NOS terminator, the opposite was found when paired with EU: the enhancement provided by 35S‐EU was significantly lower than EU‐35S (Figure 2). Furthermore, addition of BDB501 to EU resulted in a slight decrease in expression (Figure 2). Therefore, these results indicate that terminators placed in tandem interact either synergistically or antagonistically, in a context‐dependent manner.

**Figure 3:**
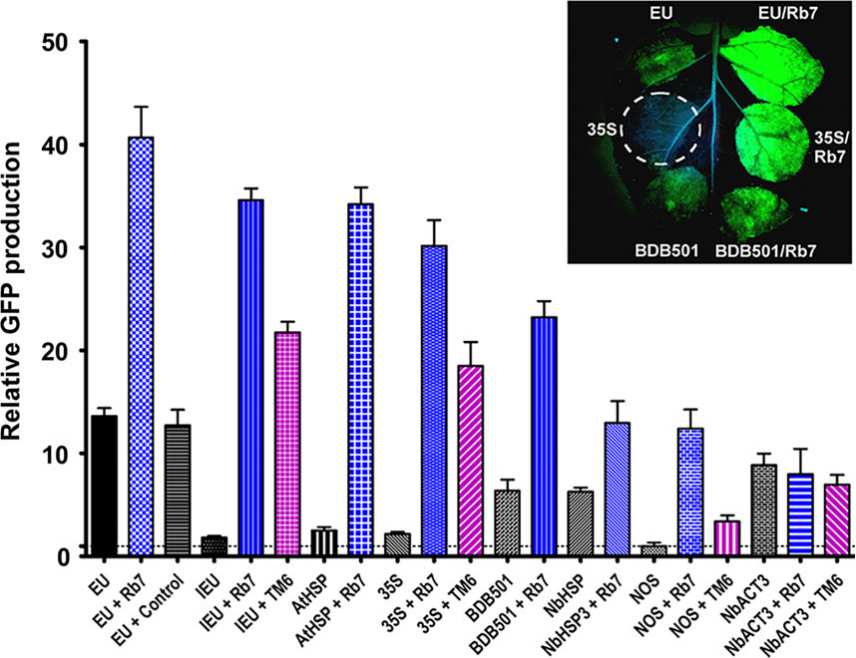
MARs strongly enhance GFP expression. Nonreplicating GFP vectors containing either the tobacco Rb7 or tobacco TM6 MAR sequences inserted 3’ of the gene terminator were agroinfiltrated into the leaves of *N. benthamiana* and evaluated for GFP production at 5 DPI. Data are means ± SE of 3–4 independently infiltrated leaves. ‘EU + Control’ indicates DNA sequence obtained from an inverted region of the Norwalk virus capsid protein coding sequence was inserted 3’ of the EU gene terminator in place of the Rb7 MAR.

Previously, we found that the 5’ and 3’ UTRs from the RNA viruses barley yellow dwarf virus (BYDV) and pea enation mosaic virus (PEMV) severely inhibited expression in *N. benthamiana* leaves using a replicating system containing the extensin terminator (Diamos *et al*., 2016). A nonreplicating expression system based on the 5’ and 3’ UTRs from cowpea mosaic virus (CMPV) was reported to enhance gene expression largely due to incorporation of the viral 3’ UTR before the NOS terminator (Meshcheriakova *et al*., 2014; Sainsbury and Lomonossoff, 2008). In this study, we evaluated virus‐derived 3’ UTRs in nonreplicating vectors. Similar to our results with replicating vectors, we found that the 3’ UTRs from PEMV, BYDV and tobacco necrosis virus‐D strongly inhibited gene expression when inserted downstream from the EU terminator, and the TMV 3’ UTR had a negligible effect on gene expression (Figure 2). In agreement with the results of Sainsbury *et al*., pEAQ‐HT‐GFP, which contains the cowpea mosaic virus 5’ and 3’ UTRs, provided a 17.1‐fold increase compared with NOS alone (Figure 3). However, this vector also contains the P19 suppressor of RNAi silencing. Coinfiltration of EU with P19 provided a 29.9‐fold increase compared with NOS (Figure 2, compare EU and EU + P19). The 3’ UTRs derived from DNA viruses also performed very well. While the BeYDV SIR showed no terminator function by itself, addition of the SIR to the 3’ end of EU nearly doubled its GFP production. Interestingly, although the intron‐containing extensin terminator (IEU) performed very poorly on its own compared with the intronless version (EU), addition of the BeYDV SIR completely negated the detrimental effect of the intron (Figure 2, compare IEU + SIR and EU + SIR). Addition of the 35S terminator to IEU also greatly enhanced expression; however in this case, the total yield was lower than the comparable vector with the intron removed (Figure 2, compare IEU + 35S and EU + 35S). These results indicate that viral 3’ flanking regions have potential to strongly increase gene expression when inserted downstream from the gene terminator.

### Matrix attachment regions are potent enhancers of transient expression

While MAR have been widely used in transgenic expression systems, there are few reports of their use in transient expression systems. We found that the tobacco Rb7 MAR strongly enhanced transient expression in a replicating geminiviral transient expression system when placed downstream from the gene terminator (Diamos *et al*., 2016). To more fully characterize the potential for MAR to function in transient expression systems, the tobacco Rb7 and TM6 MAR were inserted into nonreplicating GFP expression vectors in combination with eight different gene terminators.

Insertion of the Rb7 MAR downstream from the EU terminator resulted in a striking threefold enhancement of GFP production (40‐fold compared with NOS alone), exceeding the best double terminator configuration (Figure 3, compare EU and EU + Rb7). Interestingly, similar to the SIR, the Rb7 MAR also dramatically improved production of the otherwise weak IEU terminator, increasing expression 19‐fold, bringing it nearly on par with the EU‐Rb7 vector (Figure 3, compare IEU, IEU + Rb7 and EU + Rb7 and Figure S1A). To verify that the observed enhancement was unique to the Rb7 MAR, a control DNA sequence of similar size, derived from a synthetic norovirus capsid protein coding sequence, was instead inserted downstream of GFP and found to provide no significant difference in GFP production (Figure 3, compare EU and EU + Control). Inspection of the AT‐rich Rb7 MAR sequence reveals many terminator‐like elements; however, in the absence of a terminator, no detectable GFP activity was produced using the MAR as the sole 3’ region, indicating that it does not act as a fully functional terminator (Figure S1A). Consistent with our findings in replicating systems, positioning of the Rb7 MAR 5’ of the promoter had no effect on gene expression (Figure S1A).

We further found that Rb7 MAR provided a large enhancement when used in conjunction with the 35S (13.8‐fold), AtHSP (13.6‐fold), NOS (12‐fold), BDB501 (3.6‐fold) or NbHSP (twofold) terminators (Figure 3). In the absence of the Rb7 MAR, the NbHSP terminator provided nearly threefold more expression than the 35S terminator. However, upon addition of the MAR, the 35S/Rb7 combination provided double the expression of NbHSP/Rb7 (Figure 3). Interestingly, while the Rb7 MAR substantially enhanced seven of the eight terminators tested, the NbACT3 terminator was unaffected by addition of the Rb7 MAR.

The tobacco TM6 MAR reportedly exceeded the enhancing effect of the Rb7 MAR in transgenic tobacco (Ji *et al*., 2013). To test the TM6 MAR in our transient expression system, the full sequence was cloned from tobacco plants and inserted in place of the Rb7 MAR. The TM6 MAR enhanced GFP production when paired with the 35S, NOS or IEU terminators, but not with the NbACT3, similar to our findings for the Rb7 MAR (Figure 3, purple bars). However, the magnitude of the enhancement provided by the TM6 MAR was significantly less than that of the Rb7 MAR in all combinations tested (Figure 3, Figure S1A).

Using deletion studies, we investigated which regions of the 1193‐bp Rb7 MAR were responsible for the observed enhancement. Deletion of nucleotides 144–1193 or 437–1193 eliminated the enhancing effect of the Rb7 MAR; however, deletion of nucleotides 1–144, 144–437, 1–437, 421–730 or 1–730 did not impair MAR activity (Figure S1B). In fact, a small but repeatable increase in the enhancement provided by the Rb7 MAR was observed upon deletion of nucleotides 1–730 (Figure S1B). These data indicate that a relatively short region at the 3’ end of the Rb7 MAR is responsible for all of the observed enhancement in this system.

### Synergistic enhancement of combined 3’ flanking regions

**Figure 4:**
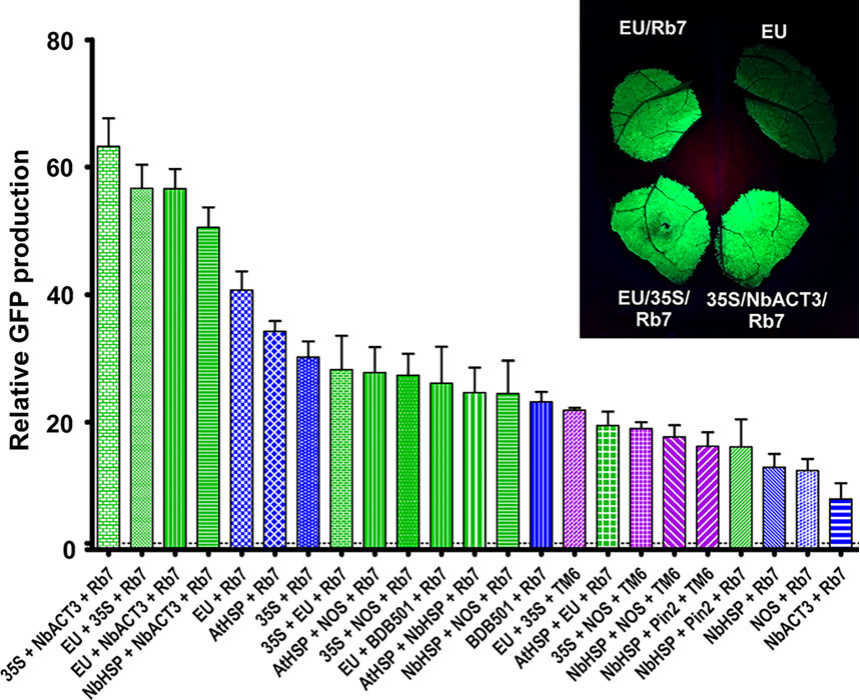
Combined 3’ UTRs strongly enhance GFP expression. Nonreplicating GFP vectors with combined terminators were created, agroinfiltrated into the leaves of *N. benthamiana* and evaluated for GFP production at 5 DPI. Data are means ± SE of 3–4 independently infiltrated leaves. Green bars indicate double terminators combined with Rb7 MAR; purple bars indicate double terminators combined with TM6 MAR; blue bars indicate single terminators combined with Rb7 MAR.

We investigated the potential for double terminators and the Rb7 or TM6 MAR to further increase gene expression when used in combination. Addition of the Rb7 MAR to the EU‐35S double terminator significantly increased the expression provided by the double terminator alone (2.4‐fold), and by either individual terminator with or without Rb7 MAR (Figure 4, compare EU + Rb7, 35S + Rb7, EU + 35S + Rb7 and Figure 3 EU + 35S). This represents a 56.7‐fold total increase compared with the NOS terminator. However, the fold increase provided by addition of the Rb7 MAR to EU‐35S was smaller than the increase provided by addition of Rb7 to either EU or 35S alone (Figure S1). Interestingly, despite the failure of the Rb7 MAR to enhance expression of the NbACT3 terminator by itself, the 35S‐NbACT3 double terminator was further enhanced by 2.4‐fold upon addition of the Rb7 MAR, more than doubling the expression provided by 35S‐Rb7 (Figure 4). EU‐NbACT3, the highest expressing double terminator, was also improved by 1.5‐fold upon addition of the Rb7 MAR.

While the 35S and NOS terminators were substantially improved when combined in either orientation (Figure 3), and while addition of the Rb7 MAR to the 35S‐NOS double terminator provided a further 1.9‐fold increase in expression (compare Figure 4 35S + NOS + Rb7 to Figure 3 35S + NOS), the total yield of 35S‐NOS‐Rb7 was no better than the single terminator construct 35S‐Rb7 (Figure 4, compare 35S + NOS + Rb7 and 35S + Rb7). Similarly, although we observed a large synergy between the Rb7 MAR and the individual terminators AtHSP, EU, BDB501 and 35S, and although the expression provided by the double terminators AtHSP‐EU, 35S‐EU and EU‐BDB501 was enhanced by addition of the Rb7, all performed worse than EU‐Rb7. Furthermore, one double terminator combination was not improved at all by the presence of a MAR. Addition of the Rb7 MAR to NbHSP‐Pin2 resulted in a small 1.1‐fold increase, which was not statistically significant (compare Figure 4 NbHSP + Pin2 + Rb7 to Figure 3 NbHSP + Pin2).

### Evaluation of combined 3’ flanking regions in a replicating system

**Figure 5:**
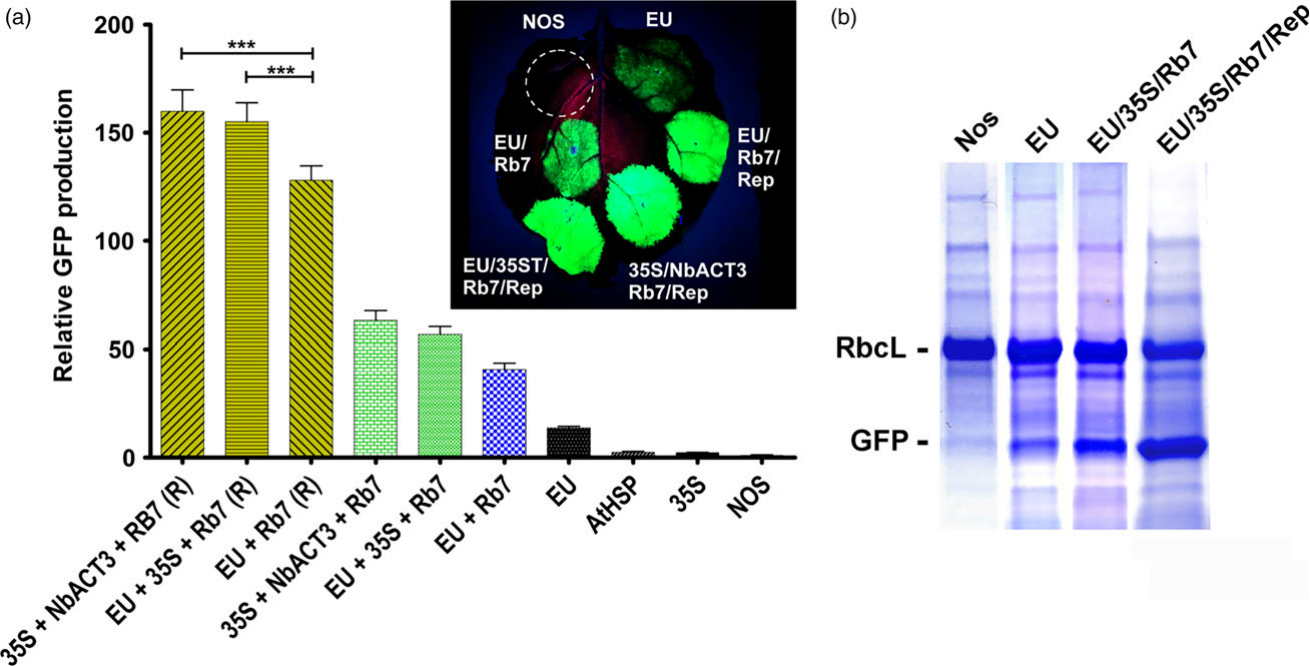
Evaluation of combined 3’ UTRs in replicating vectors. Replicating vectors containing elements of bean yellow dwarf virus (Diamos *et al*., 2016) were constructed with various combined 3’ flanking regions, agroinfiltrated into the leaves of *N. benthamiana* and evaluated for GFP production. (a) Data are means ± SE of 3–4 independently infiltrated leaves. ‘(R)’ indicates replicating geminiviral vector. (b) SDS‐PAGE gels showing GFP expression with the indicated constructs. RbcL; the large subunit of Rubisco .***P < 0.01

Previously, we reported a plant transient expression system based on the geminivirus bean yellow dwarf virus, which enhances gene expression by increasing accumulation of DNA copies of the gene of interest (Huang *et al*., 2009). We found that expression was substantially increased by insertion of the extensin terminator and the Rb7 MAR, among other modifications (Diamos *et al*., 2016). To evaluate the potential of combined 3’ UTRs to function in this system, several of the best performing 3’ UTR combinations were cloned into geminiviral vectors expressing GFP. The geminiviral vector containing EU‐Rb7 enhanced expression 3.1‐fold more than the nonreplicating vector (Figure 5A). Similarly, the two best nonreplicating vectors containing EU‐35S‐Rb7 and 35S‐NbACT3‐Rb7 were further enhanced 2.7‐fold and 2.5‐fold, respectively, when placed in geminiviral vectors (Figure 5A). Similar to their nonreplicating counterparts, in geminiviral vectors, the 35S‐EU‐Rb7 and 35S‐NbACT3‐Rb7 combinations increased GFP production by up to 20% compared with a replicating vector containing only EU‐Rb7 (Figure 5A). The total GFP expression of the best construct is estimated to be ~50% total soluble protein, or 3–5 g per kg leaf fresh weight (Figure 5B).

### Gene‐specific and plant‐specific activity of single and combined 3’ flanking regions

**Figure 6:**
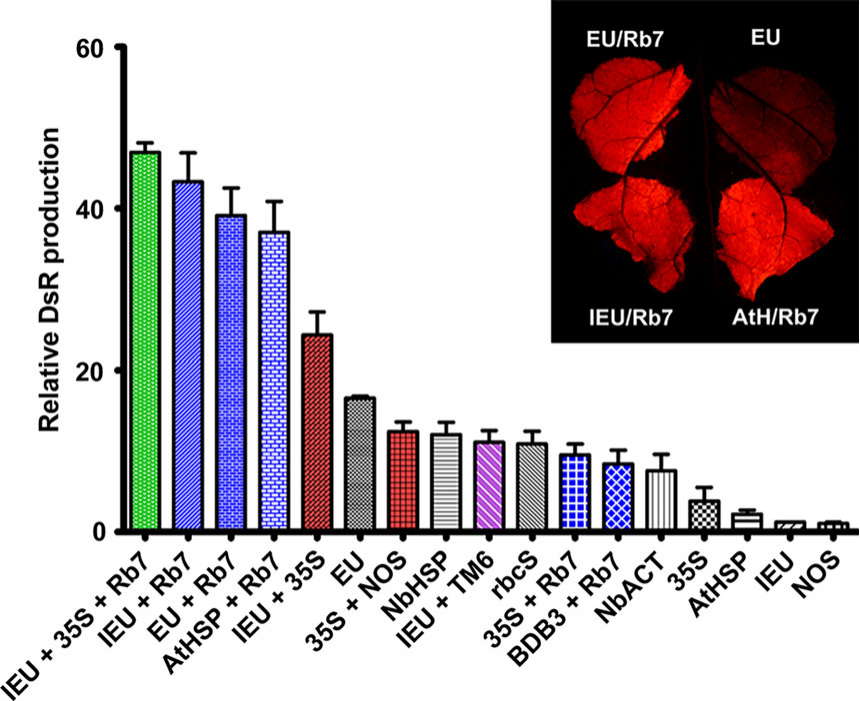
Comparison of selected 3’ UTRs expressing DsRed. Nonreplicating vectors were constructed with single, double or MAR‐containing terminators downstream from the DsRed gene and agroinfiltrated into the leaves of *N. benthamiana*. DsRed production was evaluated at 5 DPI by SDS‐PAGE and UV fluorescence. Data are means ± SE of 3–4 independently infiltrated leaves.

To determine whether the identified terminators performed similarly with a reporter gene other than GFP, vectors containing a variety of individual or combined terminators were constructed with the DsRed gene replacing the GFP gene. DsRed shares no sequence homology with GFP. For single terminators, the extensin terminator provided the highest level of gene expression (Figure 6), in agreement with our data with GUS or Norwalk virus capsid protein (Rosenthal *et al*., 2018). Other terminators performed similarly as when paired with GFP; however, some small differences were observed, such as the increased activity of NbHSP and 35S with DsRed compared with NOS (Figure 6). The IEU‐35S double terminator substantially exceeded the DsRed production of EU alone; however, the 35S‐NOS double terminator performed substantially worse with DsRed than with GFP (Figure 6). The use of the Rb7 MAR strongly enhanced DsRed expression with most terminators, and the enhancement provided by Rb7 exceeded that of TM6 in the one case tested (Figure 6). Interestingly, while 35S‐Rb7 performed very well with GFP, it did not with DsRed.

**Figure 7:**
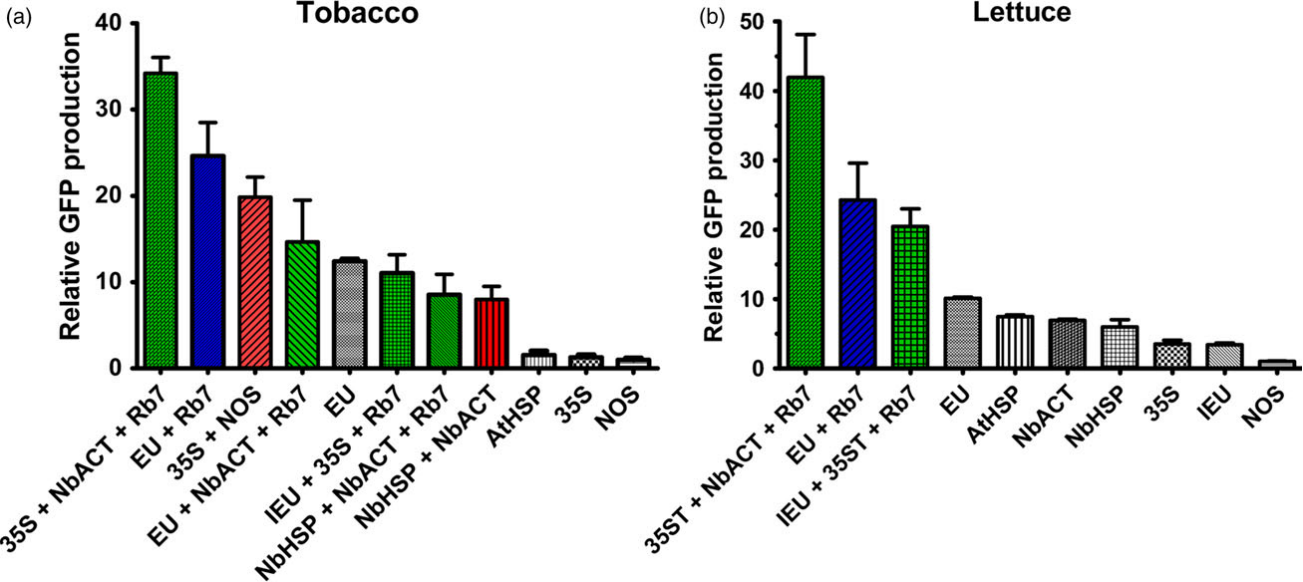
Comparison of selected 3’ UTRs in tobacco and lettuce. Nonreplicating vectors were constructed with single, double or MAR‐containing terminators downstream from the GFP gene and agroinfiltrated into the leaves of either tobacco (a) or lettuce (b) plants. GFP production was evaluated at 5 DPI by UV fluorescence and SDS‐PAGE. Data are means ± SE of 3–4 independently infiltrated leaves.

The functionality of genetic elements often varies among species. To assess the generality of these results in other plant systems, a subset of 3’ UTRs were tested in tobacco (*N. tabacum*) and lettuce. Similar to our results in *N. benthamiana*, in both tobacco and lettuce plants, GFP gene expression with EU was >10‐fold higher than with NOS, and EU exceeded all other single terminators tested (Figure 7). While EU performed substantially better than all other single terminators in its native tobacco, the AtHSP, NbHSP and NbACT3 performed nearly as well as EU in lettuce (Figure 7). Addition of the Rb7 MAR strongly enhanced GFP expression when added to EU in tobacco and lettuce, and the 35S‐NbACT3‐Rb7 combination, which performed very strongly in *N. benthamiana*, also did so in tobacco and lettuce (Figure 7). Despite its strong performance in *N. benthamiana*, IEU‐35S‐Rb7, which contains the extensin intron, had much lower expression in tobacco (Figure 7). Similarly, NbHSP‐NbACT3 with or without Rb7 MAR performed substantially worse in tobacco than in *N. benthamiana* (Figure 5, Figure 7).

## Discussion

Previously, we identified the tobacco extensin terminator as a potent enhancer of gene expression in replicating or nonreplicating systems (Diamos *et al*., 2016; Rosenthal *et al*., 2018). In this study, to more broadly assess the potential of 3’ flanking regions to enhance gene expression in plant systems, we systematically compared a diverse set of terminators from various plant and viral sources. Narsai *et al*. (2007) reported a genomewide analysis of mRNA stability in *A. thaliana*, showing that characteristic 3’ UTR motifs are enriched in long‐lived or short‐lived transcripts. To rationally derive putative terminator candidates with potential to enhance gene expression, we identified *N. benthamiana* homologs of two highly stable *A. thaliana* transcripts: an 18.8 kDa class II heat‐shock protein gene and an actin‐like gene. Both terminators outperformed all of those frequently used previously (Figure 1B). As we tested only two terminators identified in this manner, we suspect that other candidates can be discovered. Overall, we found that 12 terminators exceeded the performance of 35S or NOS.

Highly expressed genes from strong promoters are targets for RNA silencing (Que *et al*., 1997; Schubert *et al*., 2004), mediated by RDR6 (Béclin *et al*., 2002). Luo and Chen (2007) demonstrated that improperly terminated mRNAs result in RDR6‐mediated silencing of the transgene, and that the use of a 35S‐NOS double terminator reduced this effect while enhancing GUS expression is transgenic *A. thaliana*. Beyene *et al*. (2011) also found a large enhancing effect of a 35S‐NOS double terminator in several plant species. While the intronless EU terminator by itself was significantly better than all 19 other individual terminators tested, we identified eight double terminators that significantly exceeded the performance of EU alone, seven of which significantly outperformed the 35S‐NOS double terminator (Figure 2). We found that nearly every combination outperformed either individual terminator alone, showing that tandem‐linked terminators have excellent potential to enhance gene expression. Interestingly, in both tested cases, reversal of the position of the two terminators resulted in a substantial difference in expression, indicating that the observed enhancement does not arise entirely from the individual action of the two terminators, but rather on a synergistic interaction between the two terminators, which depends in part on their relative position. Furthermore, when positioned upstream from NOS, the 35S terminator performed strongly; however, the opposite was observed when paired with EU: expression was enhanced by 50% when 35S was instead positioned downstream from EU. These results indicate that the optimal position for a given terminator depends on the individual terminator with which it is paired. Further work is needed to study the individual mechanisms underlying these differences.

The 3’ flanking regions from RNA viruses contain many mechanisms to enhance mRNA stability or increase translation (Fan *et al*., 2012; Simon and Miller, 2013). However, when expressed in the plant nucleus, these 3’ regions may contain cryptic splice sites and other detrimental sequences. Most of the RNA virus‐derived 3’ flanking regions we tested were poorly functional when transiently expressed in *N. benthamiana* leaves, except for those derived from cowpea mosaic virus. The 5’ and 3’ UTRs from cowpea mosaic virus were reported to be potent enhancers of protein expression (Meshcheriakova *et al*., 2014; Sainsbury and Lomonossoff, 2008). In general agreement, we found pEAQ‐HT‐GFP, which contains the NOS terminator and the cowpea mosaic virus UTRs, enhanced GFP expression 17.1‐fold compared with NOS alone. However, the cowpea mosaic virus vector pEAQ‐HT‐GFP also contains the P19 suppressor of RNA silencing which likely enhances RNA stability (Sainsbury *et al*., 2009), making direct comparisons to other 3’ UTRs difficult. While pEAQ‐HT‐GFP provided 20% more GFP than the extensin terminator alone, it provided 40% less when extensin was also supplemented with P19 (Figure 2). We also found that other highly expressing terminator combinations identified here were also strongly enhanced by addition of P19 (data not shown). As pEAQ‐HT‐GFP contains the relatively weak NOS terminator, we suspect the cowpea mosaic virus flanking regions may perform better with a stronger terminator, unless some particular synergy exists with NOS.

Flanking regions derived from nucleus‐adapted DNA viruses, such as the geminiviruses, were found to be potent enhancers of gene expression, especially when used in conjunction with a functional terminator. The short 200‐bp SIR from bean yellow dwarf virus showed no terminator activity by itself, but was found to strongly increase gene expression when used in conjunction with the extensin terminator, on par with the best double terminator combinations tested. However, extending the SIR to include upstream and downstream coding sequence from the BeYDV coat and rep proteins showed that it also has strong terminator function on its own. Similar results were obtained with 3’ UTRs obtained from bean dwarf mosaic virus. These results highlight the influence of the upstream gene coding sequence on 3’ UTR function. Further work is needed to better characterize the enhancing potential of geminiviral 3’ UTRs and to determine whether the observed enhancing effect of the SIR is terminator‐specific.

Although MAR were previously used in transgenic systems, we found the tobacco Rb7 MAR substantially improved gene expression using a geminiviral transient expression system (Diamos *et al*., 2016). Only a small percentage of T‐DNA delivered by agrobacterium undergoes chromosomal integration, while the majority is transiently transcribed in the nucleus. It has been shown that the agrobacterium proteins VirE2, which coats the T‐DNA, and VirD2, which attaches to 5’ end of the T‐DNA and mediates nuclear entry, both associate with cellular histones (Lacroix *et al*., 2008; Wolterink‐van Loo *et al*. 2015). As MAR are thought to influence chromatin structure, the association of T‐DNA with histones suggests a possible mechanism by which MAR function in vectors delivered by agroinfiltration. Here, we find that both the tobacco Rb7 and TM6 elements greatly enhance transient gene expression in agroinfiltrated leaf tissue (Figure 3). The effect of the Rb7 MAR varies in a promoter‐dependent manner (Mankin *et al*., 2003). Similarly, we find that the effect of the MAR also varies in a terminator‐dependent manner. EU was the strongest individual terminator, and EU‐Rb7 was the strongest MAR combination, exceeding the best double terminator by over 50%. However, while NbACT3 was the second strongest individual terminator, NbACT3‐Rb7 was the lowest expressing MAR combination. Interestingly, we saw a similar effect when NbACT3 was paired with the TM6 MAR, suggesting that some enhancing activity present in both MAR is not active when paired with the NbACT3 region. All other terminators greatly benefited from addition of the Rb7 or TM6 MARs, although the magnitude of the enhancement varied in a manner that was not correlated to the individual expression level mediated by each terminator alone.

Ji *et al*. (2013) found that the TM6 MAR enhanced GUS expression at a level greater than the Rb7 MAR in transgenic tobacco. However, we consistently found that the Rb7 MAR increased transient expression more than the TM6 MAR. This observed discrepancy could be due to different expression systems, or different reporter genes. We found that the entire enhancing activity of the Rb7 MAR resides in a 463‐bp region at its 3’ end. Although a detailed characterization of the functional regions of the Rb7 MAR has not been reported, the region we found to be dispensable includes several AT‐rich regions, a matrix attachment recognition sequence motif and a topoisomerase II binding site, all of which were previously suspected to play a role in MAR function (Allen *et al*., 1996). Ji *et al*. (2013) found that deletion of similar MAR elements substantially reduced the enhancing effect of TM6. Additionally, it has been reported that the TM2 MAR functions best when placed 5’ of the gene of interest (Zhang *et al*., 2009), whereas we found no effect of 5’ insertion of the Rb7 MAR. As MAR are thought to contain multiple active regions responsible for their enhancing function, there may be differences in the key functional regions of the Rb7, TM2 and TM6 MARs, making direct comparison difficult. Alternatively, while Rb7 and TM6 are both clearly active in our transient expression system, the mechanisms by which expression is enhanced may differ between transient and transgenic systems. Further studies are needed to resolve these discrepancies.

Previously, we found that combining optimized 5’ UTRs and the Rb7 MAR resulted in a synergistic enhancement of gene expression (Diamos *et al*., 2016). Other studies obtained favourable results by duplicating or combining highly functional genetic elements, such by tandem‐linking TM2 MAR (Zhang *et al*. 2009) or combining the 5’ UTR from alcohol hydrogenase and the AtHSP terminator (Limkul *et al*., 2015). Here, we find that combining double terminators with the Rb7 MAR enhanced gene expression more than either component by itself in some, but not most, cases (Figure 4). Particularly high synergy was observed between the Rb7 MAR and the EU‐35S, 35S‐NbACT3 and EU‐NbACT3 double terminators, reaching very high expression levels of up to 60‐fold more than the NOS terminator alone.

We saw variable effects when combining double terminators with MAR. While 35S‐NOS was a relatively strong double terminator, it had little synergy when combined with Rb7. Similarly, while the AtHSP terminator had high synergy with the Rb7 MAR, double terminators containing AtHSP did not improve expression compared with AtHSP‐Rb7 alone (Figure 4). As all AtHSP double terminators tested in this study had AtHSP positioned as the upstream terminator, it is possible reversing terminator positions may result in better performance. Notably, EU‐35S‐Rb7 was one of the best combinations tested, but the reversed 35S‐EU‐Rb7 had a 50% reduction in expression (Figure 4).

We have created a replicating transient expression system based on the geminivirus bean yellow dwarf virus, which amplifies the gene of interest to high copy number in the plant nucleus (Huang *et al*., 2009, 2010). By incorporating optimized 5’ and 3’ UTRs with other modifications, we have used this system to produce vaccine antigens and pharmaceutical proteins at levels greater than or similar to the highest levels reported in plant‐based systems (Diamos *et al*., 2016). Here, we find that gene expression with the double terminator and MAR constructs 35S‐NbACT3‐Rb7 and EU‐35S‐Rb7 is improved by ~2.5‐fold when placed in a replicating vector, a 20% increase compared with the best replicating construct containing only a single terminator and MAR (Figure 5A). This represents a more than 150‐fold increase compared with the original NOS vector alone, providing an estimate yield of around 40%–60% total soluble protein or 4–5 mg recombinant protein per kg of leaf tissue (Figure 5B), which appears to approach the theoretical limit achievable in a plant‐based system.

The upstream gene coding sequence has been shown to interact with the 3’ UTR. The NOS terminator contains a cryptic polyadenylation site that requires an upstream element to be present for its function (Sanfaçon and Hohn, 1990; Sanfaçon *et al*., 1991). We found that the intergenic regions of bean yellow dwarf virus and bean dwarf mosaic virus both require upstream coat protein coding sequence for terminator function (Figure 1B). These results indicate that 3’ UTRs may perform differently in the context of different genes of interest. Using DsRed as an alternative reporter gene to GFP with no shared homology, we found that most single or combined 3’ UTRs performed similarly relative to one another, with a few notable exceptions. The 35S‐NOS and 35S‐Rb7 3’ UTRs both performed substantially worse with DsRed than they did with GFP, however, both still performed better than 35S alone. Despite these combinations performing worse, the 35S terminator alone performed better with DsRed than with GFP, and combinations with the 35S terminator positioned as the second terminator were still highly functional (Figure 6).

Lettuce has been shown to be a promising plant system capable of rapidly producing recombinant proteins (Chen *et al*., 2016; Lai *et al*., 2012). To further investigate the generality of our results, we also tested a variety of 3’ UTRs in tobacco and lettuce. As with *N. benthamiana*, EU was the best individual terminator in lettuce. Further, combined terminators containing the Rb7 MAR substantially outperformed any individual terminator tested (Figure 7). However, a few of the terminator combinations that performed very well in *N. benthamiana* performed relatively poorly in either lettuce or tobacco. As we did not test every identified combination with multiple genes or other plant systems, it is likely that other gene‐specific or plant‐specific effects exist that we did not discover here. Overall, our data suggest that the optimal terminator for a given system must be determined empirically. However, the potent enhancing effect of the intronless EU terminator has been demonstrated with GFP, DsRed, GUS and Norwalk virus capsid protein, in *N. benthamiana*, lettuce and tobacco (Figure 1B, Figure 6, Figure 7 and Rosenthal *et al*., 2018), indicating that the terminator is intrinsically highly active in many gene contexts. Additionally, our results clearly demonstrate that combining terminators is a highly effective strategy to improve gene expression in a variety of systems.

In conclusion, we have identified a diverse set of gene terminator regions that greatly exceed the gene expression provided by the most commonly used terminators in *N. benthamiana*, tobacco and lettuce leaves. The intronless tobacco extensin terminator is a uniquely potent enhancer of gene expression. In nearly every case tested, double terminators outperformed either individual terminator alone, often exceeding the gene expression of the best individual terminators by more than twofold. We find that MAR, especially the 3’ end of the Rb7 MAR, are strong enhancers of transient gene expression, and when combined with double terminators, synergistically enhance expression. Incorporating these combined terminators into a replicating geminiviral expression system has allowed us to produce recombinant proteins compared with the highest levels ever reported in a plant‐based system. We anticipate that the 3’ UTR combinations identified here to have broad potential to improve other DNA‐based plant expression systems. The 3’ region sequences studied here are available in the supplemental material.

## Experimental procedures

### Vector construction

An agrobacterium T‐DNA binary vector containing the 35S promoter, tobacco mosaic virus 5’ UTR, GFP gene and full‐length tobacco extensin terminator was constructed by 3‐fragment ligation: the vector backbone from pPS1 (Huang and Mason, 2004) was obtained by XhoI‐EcoRI digestion; the TMV 5’ UTR‐GFP fragment was excised from pBYR2e‐GFP (Diamos *et al*., 2016) by XhoI‐SacI digestion; and the intronless extensin terminator was excised from pBY‐GFP212 (Diamos *et al*., 2016). The resulting vector, pPS‐OGFP‐EU, was used to construct single and double terminator constructs. The DsRed gene was amplified from pBYDsRed (Huang *et al*., 2010) with primers DsR‐Xba‐F and VspHT, digested XbaI‐SacI and inserted into pPS‐OGFP‐EU to create pPS‐ODsR‐EU. Terminators were amplified by PCR using primers (Table S1) designed to insert SacI and EcoRI restriction sites at its 5’ and 3’ ends, respectively, digested SacI‐EcoRI and ligated into pPS‐OGFP digested likewise. The pinII, NOS and rbcS 3’ regions were obtained from pHB114 (Richter *et al*., 2000), pHB103 (Richter *et al*., 2000) and pRTL2‐GUS (Carrington *et al*., 1991), respectively, by SacI‐EcoRI digestion and cloned into pPS‐OGFP digested likewise. Further construction details are available in Methods S1, and a complete list of 3’ UTR sequences can be found in the supplemental ‘3’ Region Sequences.’

### Growth and agroinfiltration of plant leaves

Binary vectors were separately introduced into *Agrobacterium tumefaciens* GV3101 by electroporation. The resulting strains were verified by restriction digestion or PCR, grown overnight at 30 °C and used to infiltrate leaves of 5‐ to 6‐week‐old *Nicotiana benthamiana*, tobacco (*N. tabacum*) or lettuce (*Lactuca sativa* ‘Black Seeded Simpson’) maintained at 23–25 °C. Briefly, the bacteria were pelleted by centrifugation for 5 min at 5000 *g* and then resuspended in infiltration buffer (10 mm 2‐(N‐morpholino)ethanesulfonic acid (MES), pH 5.5 and 10 mm MgSO4) to OD600 = 0.2. The resulting bacterial suspensions were infiltrated using a syringe without needle into leaves through a small puncture (Huang and Mason, 2004). Plant tissue was harvested 5 days postinfiltration (DPI). Leaves producing GFP were photographed under UV illumination generated by a B‐100AP lamp (UVP, Upland, CA).

### Protein extraction and fluorescence analysis

Total protein extract was obtained by homogenizing agroinfiltrated leaf samples with 1:5 (*w:v*) ice‐cold extraction buffer (25 mm sodium phosphate, pH 7.4, 100 mm NaCl, 1 mm EDTA, 0.1% Triton X‐100, 10 mg/mL sodium ascorbate, 0.3 mg/mL PMSF) using a Bullet Blender machine (Next Advance, Averill Park, NY) following the manufacturer's instruction. To enhance solubility, the homogenized samples were end‐over‐end mixed at room temperature or 4 °C for 30 min. The crude plant extract was clarified by centrifugation at 13 000 ***g*** for 10 min at 4 °C. Protein concentration of clarified leaf extracts was measured using a Bradford protein assay kit (Bio‐Rad, Hercules, CA) with bovine serum albumin as standard. For SDS‐PAGE, clarified plant proteins extract were mixed with sample buffer containing a final concentration of 50 mm Tris‐HCl, pH 6.8, 2% SDS, 10% glycerol, 0.02% bromophenol blue, and separated on 4%–15% polyacrylamide gels (Bio‐Rad). For GFP or DsRed fluorescence, PAGE gels were visualized under UV illumination (365 nm) and stained with Coomassie stain (Bio‐Rad) following the manufacturer's instructions. The fluorescent band corresponding to GFP or DsRed was analysed using ImageJ software to quantify the band intensity using native plant protein bands as an internal loading control.

## Supporting information


**Figure S1** Characterization of Rb7 and TM6 MARs.

**Methods S1** Additional vector construction information.
**Table S1** Table of oligonucleotides used in this study.
