# Supplementary figures and images for: Chimeric 3’ flanking regions strongly enhance gene expression in plants
Source: Plant Biotechnol J. 2018 May 21;16(12):1971–82. doi: 10.1111/pbi.12931 (PMC6230951; doi:10.1111/pbi.12931)

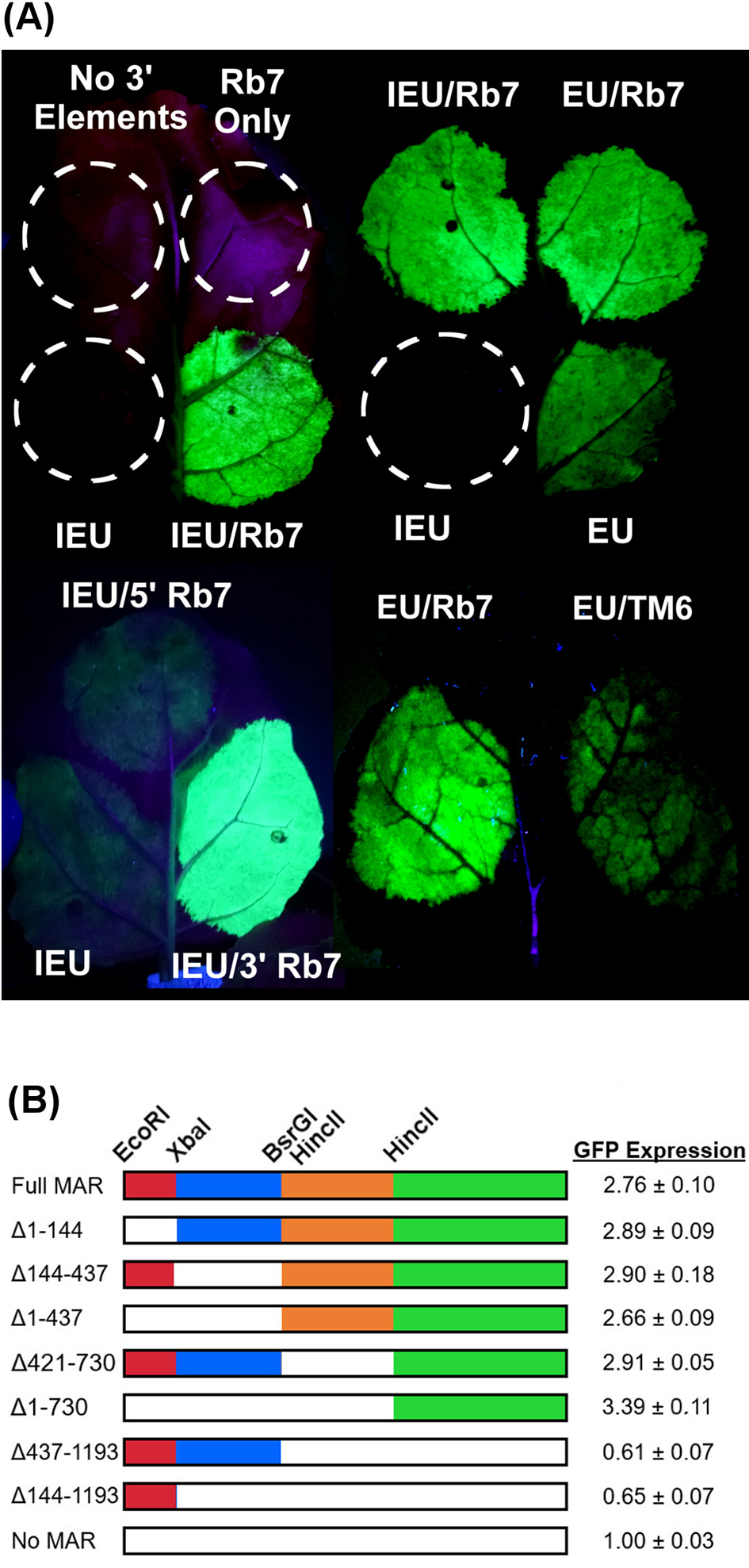

Supplement: Supplementary file 1 — Figure S1 Characterization of Rb7 and TM6 MARs. [file PBI-16-1971-s002.tif]
