# Supplementary material for: Chimeric 3’ flanking regions strongly enhance gene expression in plants
Source: Plant Biotechnol J. 2018 May 21;16(12):1971–82. doi: 10.1111/pbi.12931 (PMC6230951; doi:10.1111/pbi.12931)
Supplement: Supplementary file 2 — Methods S1 Additional vector construction information. Table S1 Table of oligonucleotides used in this study. [file PBI-16-1971-s001.docx]

### Methods S1. Vector Construction Details

The NbHSP (homolog of At5g12020) and NbACT3 (homolog of At5g09810) terminators were identified using the Sol Genomics Network *N. benthamiana* draft genome (Fernandez-Pozo *et al.*, 2015). Primers (Table S1) specific for NbHSP and NbACT3 designed to introduce SacI and EcoRI sites were used to amplify the downstream segments of each gene from *N. benthamiana* genomic DNA. The PCR products were digested SacI-EcoRI and inserted into pPS-OGFP-EU digested likewise.

For double terminator constructs, the upstream segment was amplified by PCR using primers (Table S1) designed to insert a SacI site at the 5’ end, and BsaI site at the 3’ end. The downstream segment was amplified with a BsaI site at the 5’ end designed to generate compatible overhang with the upstream BsaI site, and an EcoRI site at the 3’ end. The final construct was assembled by 3-fragment ligation: pPS-OGFP-EU digested SacI-EcoRI, the upstream segment digested SacI-BsaI, and the downstream segment digested BsaI-EcoRI.

For MAR constructs, the tobacco Rb7 or TM6 MAR was inserted downstream from the terminator. First, the Rb7 MAR was inserted into pPS-OGFP-EU by three fragment ligation: pPS-OGFP-EU was digested PvuI-SphI to obtain the vector fragment; pPS-OGFP-EU was digested PvuI-EcoRI to obtain the GFP cassette; and pBYR2e-MRtxGM (Diamos et al., 2016) was digested EcoRI-SphI to obtain the Rb7 MAR fragment. The resulting vector was digested KpnI-AgeI, the ends were blunted with Klenow fragment DNA polymerase, and the vector fragment was self-ligated to yield pPS-OGFPM-EU. The TM6 MAR (genbank accession KC555564) was PCR amplified from tobacco genomic DNA using primers TM6-EcoRI-F and TM6-KpnI-R (Ji *et al.*, 2013), digested EcoRI-KpnI, and inserted into pUC19. The EcoRI-AvrII fragment containing the TM6 MAR was then excised and inserted into pPS-OGFPM-EU digested likewise to yield pPS-OGFPT-EU. Single or double terminators were inserted into pPS-OGFPM-EU or pPS-OGFPT-EU by SacI-EcoRI digestion as described for pPS-OGFP-EU. For Rb7 deletion mutants, native restriction sites were used as shown in Fig. 5. After digestion with each respective enzyme, the ends were blunted with Klenow fragment DNA polymerase, and self-ligated.

### Table S1. Oligonucleotides used in this study

| TM6-EcoRI-F | TCCGAATTCTAATATTTAGAAATTTAATTAACATAACCAAGG |
| --- | --- |
| TM6-KpnI-R | CTGGTACCGACATCCTAGGTTCAATCAAAT |
| DsR-Xba-F | GAGTCTAGAACATGGTGCGCTCCTCC |
| VspHT | TGAATAGTGCATATCAGCATACCTTA |
| EU-Bsa-F | AGGGTCTCGGCTCAAAGCAGAATGCTG |
| EU-Bsa-R | AAGGTCTCGGAGCGTCATAACTGTAGAAATGATTCC |
| BDB-Bsa-F | GGGTCTCGGCTCTGACAACATCAGCAAG |
| NbHSP-Bsa-F | AGGGTCTCGGCTCACTGAGGAAATATATAGACAAATTAAG |
| 35S-Bsa-R | AAGGTCTCGGAGCGTCACTGGATTTTGGTTTTAGG |
| NbHSP-Bsa-R | AAGGTCTCAGAGCTCCCAAAGGAAACTATGTGTAC |
| NOS-Bsa-R | AGGGTCTCGGCTCAGATCGTTCAAACATTTG |
| Pin2-Bsa-F | GAGGTCTCAGCTCGTACCCTGCAATGTGACC |
| NbACT-Bsa-F | AGGGTCTCGGCTCATACAGCATTCCCA |
| BDB501-Sac-F | CCGAGCTCTGACAACATCAGCAAGAACG |
| BDB501-Eco-R | AAGAATTCAAAGGAAACCCATAAGATGCG |
| NbHSP-Sac-F | TCGAGCTCACTGAGGAAATATATAGACAAATTAAGTTTGGTTCTATG |
| NbHSP-Eco-R | GTGAATTCGCTCCCAAAGGAAACTATGTGTACTTC |
| NbACT-Sac-F | GCGAGCTCATACAGCATTCCCAGAAAGAGAAAC |
| NbACT-Eco-R | TAGAATTCATGCTAGCTTGTTTACACCTCG |
| BDA375-Sac-F | GAGAGCTCGGAGAACGCCTTATTATTGTATATGGC |
| BDA375-Eco-R | AAGAATTCGCTCATCACTGCACTTCAAGC |
| AtHSP-Sac-F | TAGAGCTCATATGAAGATGAAGATGAAATATTTGGTGTG |
| AtHSP-Eco-R | ATGAATTCCTTATCTTTAATCATATTCCATAGTCCATACC |
| AtHSP-Bsa-R | AAGGTCTCGGAGCCTTATCTTTAATCATATTCCATAGTCCATACC |
| Rep-Sac-F | AGCGAGCTCTAATAGGTTGCCAGTCTGATTTC |
| Rep-Eco-R | CTAGAATTCTTGCCATCGTTTTGTGG |
| RepA-Sac-F | TCGGAGCTCTGAACGTGCCTCTCCTC |
| SIR-Sac-F | AAGGAGCTCTAAAATGATTATTTTATGAATATATTTCATTGTGC |
| NbACT617-EcoR | AATGAATTCGAACCCCAATTACTGGAGC |
| 35S-Bsa-F | GCGGTCTCGGCATGGTGGAGCACGA |
| NOS-Bsa-F | AGGGTCTCGGCTCAGATCGTTCAAACATTTG |
| TNVD3-Bsr-F | ATTGTACAAGTAATTGCTTTCATAGATCCGTCTTCC |
| TNVD3-Sac-R | TAGAGCTCGGGTTCCTAGAGAGATCTCTAGG |
| TMV3-Bsr-F | TATGTACAAGTAAGGTAGTCAAGATGCATAATAAATAACGGATTGTG |
| TMV3-Sac-R | TAGAGCTCTGGGCCCCTACCGGGGGTAA |
| TNVD5-F | TCGAGATACCTAACCAGTGTCTCAGTGATTAAGTAATCAGCT |
| TNVD5-R | CTAGAGCTGATTACTTAATCACTGAGACACTGGTTAGGTATC |
| PEMV5-F1 | TCGAGGGTATTTATAGAGATCAGTATGAACTGTGTCGCTAGGATCAAGCGG |
| PEMV5-F2 | TGGTTCACACCTGACTTCACCCCTGGCGAGGGCGTGAAGTCTAC |
| PEMV5-R1 | CATGGTAGACTTCACGCCCTCGCCAGGGGTGAAGTCAGGTGTGAACCACCGC |
| PEMV5-R2 | TTGATCCTAGCGACACAGTTCATACTGATCTCTATAAATACCC |
| PEMV3-Bsr-F | ATTGTACAAGTAAGGCTTCGCTTCCCGCC |
| BYDV3-Kpn-F | AAGGTACCAGTGAAGACAACACC |
| BYDV3-Sac-R | ATGAGCTCGGGTTGCCGAACTGC |

### 3’ Region Sequences:

Lower case letters indicate the 3’ flanking region sequence. Capital letters indicate extraneous sequence, such as restriction sites.

**NOS** GAGCTCagatcgttcaaacatttggcaataaagtttcttaagattgaatcctgttgccggtcttgcgatgattatcatataatttctgttgaattacgttaagcatgtaataattaacatgtaatgcatgacgttatttatgagatgggtttttatgattagagtcccgcaattatacatttaatacgcgatagaaaacaaaatatagcgcgcaaactaggataaattatcgcgcgcggtgtcatctatgttactagatcggcgatcggggctgcagGAATTC

**35S**GAGCTCgtccgcaaaaatcaccagtctctctctacaaatctatctctctctatttttctccagaataatgtgtgagtagttcccagataagggaattagggttcttatagggtttcgctcatgtgttgagcatataagaaacccttagtatgtatttgtatttgtaaaatacttctatcaataaaatttctaattcctaaaaccaaaatccagtgacGAATTC

**PinII**GAGCTCgcccggggatcctctagagtaccctgcaatgtgaccctagacttgtccatcttctggattggccaagttaattaatgtatgaaataaaaggatgcacacatagtgacatgctaatcactataatgtgggcatcaaagttgtgtgttatgtgtaattactaattatctgaataagagaaagagatcatccatatttcttatcctaaatgaatgtcacgtgtctttataattctttgatgaaccagatgcattttattaaccaattccatatacatataaatattaatcatatataattaatatcaattgggttagcaaaacaaatctagtctaggtgtgttttgctaattattgggggatagtgcaaaaagaaatctacgttctcaataattcagatagaaaacttaataaagtgagataatttacatagattgcttttatcctttgatatatgtgaaaccatgcatgatataaggaaaatagatagagaaataattttttacatcgttgaatatgtaaacaatttaattcaagaagctaggaatataaatattgaggagtttatgattattattattattttgatgttcaatgaagttttttttaatttcatatgaagtatacaaaaattcttcatagatttttgtttctatgccgtagttatctttaatatatttgtggttgaagaaatttattgctagaaacgaatggattgtcaatttttttttaaagcaaatatatatgaaattatactgtatattattttagtcatgattaaaatgtggccttaattgaatcatctttctcattcattttttcaaaagcatatcaggatgattgatatttatctattttaaaaattaatttaagggttcaaattaaatttaacttaaaagtgtcctaaccgtagttaaaggtttactttaaaaaaatactatgaaaaatctaatcttctatgaatcgacctgcagGAATTC

**rbcS**GAGCTcccaattcgccctatagtgagtcgtattacgcgcggagctttcgttcgtatcatcggtttcgacaacgttcgtcaagttcaatgcatcagtttcattgcgcacacaccagaatcctactgagtttgagtattatggcattgggaaaactgtttttcttgtaccatttgttgtgcttgtaatttactgtgttttttattcggttttcgctatcgaactgtgaaatggaaatggatggagaagagttaatgaatgatatggtccttttgttcattctcaaattaatattatttgttttttctcttatttgttgtgtgttgaatttgaaattataagagatatgcaaacattttgttttgagtaaaaatgtgtcaaatcgtggcctctaatgaccgaagttaatatgaggagtaaaacacttgtagttgtaccattatgcttattcactaggcaacaaatatattttcagacctagaaaagctgcaaatgttactgaatacaagtatgtcctcttgtgttttagacatttatgaactttcctttatgtaattttccagaatccttgtcagattctaatcattgctttataattatagttatactcatggatttgtagttgagtatgaaaatattttttaatgcattttatgacttgccaattgattgacaacatgcatcaagctatcGAATTC

**IEU**GAGCTCgaagtgacatcacaaagttgaaggtaataaagccaaattaattaagacattttcataatgatgtcaagaatgcaaagcaaattgcataactgcctttatgcaaaacattaatataatataaattataaagaactgcgctctctgcttcttattttcttagcttcatttattagtcactagctgttcagaattttcagtatcttttgatattactaagaacctaatcacacaatgtatattcttatgcaggaaaagcagaatgctgagctaaaagaaaggctttttccattttcgagagacaatgagaaaagaagaagaagaagaagaagaagaagaagaagaaaagagtaaataataaagccccacaggaggcgaagttcttgtagctccatgttatctaagttattgatattgtttgccctatattttatttctgtcattgtgtatgttttgttcagtttcgatctccttgcaaaatgcagagattatgagatgaataaactaagttatattattatacgtgttaatattctcctcctctctctagctagccttttgttttctctttttcttatttgattttctttaaatcaatccattttaggagagggccagggagtgatccagcaaaacatgaagattagaagaaacttccctcttttttttcctgaaaacaatttaacgtcgagatttatctctttttgtaatggaatcatttctacagttatgacGAATTC

**EU**GAGCTCaaagcagaatgctgagctaaaagaaaggctttttccattttcgagagacaatgagaaaagaagaagaagaagaagaagaagaagaagaagaaaagagtaaataataaagccccacaggaggcgaagttcttgtagctccatgttatctaagttattgatattgtttgccctatattttatttctgtcattgtgtatgttttgttcagtttcgatctccttgcaaaatgcagagattatgagatgaataaactaagttatattattatacgtgttaatattctcctcctctctctagctagccttttgttttctctttttcttatttgattttctttaaatcaatccattttaggagagggccagggagtgatccagcaaaacatgaagattagaagaaacttccctcttttttttcctgaaaacaatttaacgtcgagatttatctctttttgtaatggaatcatttctacagttatgacGAATTC

**NbHSP**GAGCTCactgaggaaatatatagacaaattaagtttggttctatgagttctaatttggacttaagagttgtttgaaattctattttatagtgatgcttataatgtatttggactgttttctgctgtgtgtaagaccttttggtctgtgaactggaaacatacatgaataaatttctttgaatttactggaatttttgcatcaacaaaagaaaaattgaagttactaacttgtaaatggaacaattgtaatgttaaaggatataaatatcttaatatagtgcgatacgaatcacacgaatgcaagactttctctctctgctcccgctcatgctctcggtgcatgttagctaaatatacatcggtgcatccatggcaggagcatgaggacggggatgaggaagggagtgaggagggccaaaagaagtacacatagtttcctttgggagcGAATTC

**NbACT**GAGCTCatacagcattcccagaaagagaaacagaagaaatatacaaactttcattttgagagcagcacctcgtctattgattgcagataatatgcttctcatttgtatttccttttgattatttttgtttctatccctttgtttgagtcaatctcaaatattcggtcattgttggtatgaaaaatcaagcagttcatgttaagagtcaatttaaaattaatatttttatatagagttgtatgtgaaatgatgttgtgatttggtatatatggataaagagcttgtcagttcattttggtctcatttttttggtatccaaataagaaacacaaaagggatatgtccctctactatcaaatattagttataagtattcatgttatactattcgatattttctaccccaatcgttacctatttaaaagtatttacccctccatctatcaaacccctggacccagctttcctattacatgtggcttcatcttaagcccccaaacctttttcttatttttgatttttaaaggctcatcttaaaatttattactcaaattaatacctcttaataacccacctcaaggacccagtaattaaatatccaattagctccagtaattggggttcatattagctccagtcttaaattttaaaggcgatgatcgtattcctccacttggttcatttatactcaaagaatactcaatgtctttagtgtttagataactttttgtaaatcatatagattgttttaacaaaaaacaattcaatagtagattttcacatgaaagttacataaaaattctttaaaattactttctcaaaaaattgttccaaacatattatcccacaattaaactcaatctgtttttcgaaacctaaatcaaaaccaatccaactaccttatataatatataatcaatacattgtaaagaactgcatgttcttttaaattttgggggcaaagttattccgtacgttcacacatgtactaataggaggtaataaatgatatgtgaaacaatcgaggtgtaaacaagctagcatGAATTC

**SIR**GAATTCcgagtgtacttcaagtcagttggaaatcaataaaatgattattttatgaatatatttcattgtgcaagtagatagaaattacatatgttacataacacacgaaataaacaaaaaaacacaatccaaaacaaacaccccaaacaaaataacactatatatatcctcgtatgaggagaggcacgttcagtgactcgacgattcctagGCCGGC

**SIR 3’**GAGCTCtaaaatgattattttatgaatatatttcattgtgcaagtagatagaaattacatatgttacataacacacgaaataaacaaaaaaacacaatccaaaacaaacaccccaaacaaaataacactatatatatcctcgtatgaggagaggcacgttcagtgactcgacgattcccgagcaaaaaaagtctccccgtcacacatatagtgggtgacgcaattatcttcaaagtaatccttctgttgacttgtcattgataacatccagtcttcgtcaggattccaaagaattatagaagggatcccaccttttattttcttcttttttccatatttagggttgacagtgaaatcagactggcaacctattaattgcttccacaatgggacgaacttgaaggggatgtcgtcgatgatattataggtggcgtgttcatcgtagttggtgaagtcgatggtcccgttccagtagttgtgtcgcccgagacttctagcccaggtggtctttccggtacgagttggtccgcagatgtagaggctggggtgtctgaccccagtccttccctcatcctggttagatcggccatccactcaaggtcagattgtgcttgatcgtaggagacaggatgtatgaaagtgtaggcatcgatgcttacatgatataggtgcgtctctctccagttgtgcagatcttcgtggcagcggagatctgattctgtgaagggcgacacgtactgctcaggttgtggaggaaataatttgttggctgaatattccagccattgaagctttgttgcccattcatgaggGAATTC

**SIR 5’/3’**GAGCTCcatttcatttttggctggtgtacgacaagtctcctggtgctaatgttcccagtactggggatatatttgagggaccttcgttgtttcctcataatccttggacgtggactgtgtctagggctgcttgccatcgttttgtggtgaagaaaacgtggtcatgcgtggtggagagcaatggtattgacccaaccaaaggtcaaggagctacttattatgggcctgggccttgtaatcaggttaagtcctgtaataagttctttaagagattgggtgtgtccacagagtggaaaaatagtgcaacgggtgatgttggtgatataaaggaaggagccctttacattgttggtgctccttcccaaaagtccgatgtatatgtaaatggttatttccgagtgtacttcaagtcagttggaaatcaataaaatgattattttatgaatatatttcattgtgcaagtagatagaaattacatatgttacataacacacgaaataaacaaaaaaacacaatccaaaacaaacaccccaaacaaaataacactatatatatcctcgtatgaggagaggcacgttcagtgactcgacgattcccgagcaaaaaaagtctccccgtcacacatatagtgggtgacgcaattatcttcaaagtaatccttctgttgacttgtcattgataacatccagtcttcgtcaggattccaaagaattatagaagggatcccaccttttattttcttcttttttccatatttagggttgacagtgaaatcagactggcaacctattaattgcttccacaatgggacgaacttgaaggggatgtcgtcgatgatattataggtggcgtgttcatcgtagttggtgaagtcgatggtcccgttccagtagttgtgtcgcccgagacttctagcccaggtggtctttccggtacgagttggtccgcagatgtagaggctggggtgtctgaccccagtccttccctcatcctggttagatcggccatccactcaaggtcagattgtgcttgatcgtaggagacaggatgtatgaaagtgtaggcatcgatgcttacatgatataggtgcgtctctctccagttgtgcagatcttcgtggcagcggagatctgattctgtgaagggcgacacgtactgctcaggttgtggaggaaataatttgttggctgaatattccagccattgaagctttgttgcccattcatgaggGAATTC

**BDB501**GAGCTCtgacaacatcagcaagaacgccctcctagtatattactgttggatgtcagatactatgtcaaaggcatctacttttgtatcgtttgaccttgattatatcggttgattaatgataattgtaataaaaagctattattgaactttcaattcctcaacaaagaaattattgcaacgatttgggctgataagccttacagttactatttatacactcctggacagtgtttttcactagctcgtttaattgccccatcgacatagtaatgttggattccgctctctgggcccctacaattgaggcagactcccctgggtctaagacgcttgttccaagcctgctgagatgcctatatggatgcattgcgttttccacctctgagtcggcatcggagttgctgagcccaattgtactccgtgaagcccatgattcacccggcttgatctctattgggcctggtagtccaatccttgacatggatgcgcatcttatgggtttcctttGAATTC

**AtHSP**GAGCTCatatgaagatgaagatgaaatatttggtgtgtcaaataaaaagcttgtgtgcttaagtttgtgtttttttcttggcttgttgtgttatgaatttgtggctttttctaatatcaaatgaatgtaagatctcattataatgaataaacaaatgtttctataatccattgtgaatgttttgttggatctcttctgcagcatataactactgtatgtgctatggtatggactatggaatatgattaaagataaGAATTC

**Rep**GAGCTCtaataggttgccagtctgatttcactgtcaaccctaaatatggaaaaaagaagaaaataaaaggtgggatcccttctataattctttggaatcctgacgaagactggatgttatcaatgacaagtcaacagaaggattactttgaagataattgcgtcacccactatatgtgtgacggggagactttttttgctcgggaatcgtcgagtcactgaacgtgcctctcctcatacgaggatatatatagtgttattttgtttggggtgtttgttttggattgtgtttttttgtttatttcgtgtgttatgtaacatatgtaatttctatctacttgcacaatgaaatatattcataaaataatcattttattgatttccaactgacttgaagtacactcggaaataaccatttacatatacatcggacttttgggaaggagcaccaacaatgtaaagggctccttcctttatatcaccaacatcacccgttgcactatttttccactctgtggacacacccaatctcttaaagaacttattacaggacttaacctgattacaaggcccaggcccataataagtagctccttgacctttggttgggtcaataccattgctctccaccacgcatgaccacgttttcttcaccacaaaacgatggcaaGAATTC

**RepA**GAGCTCtgaacgtgcctctcctcatacgaggatatatatagtgttattttgtttggggtgtttgttttggattgtgtttttttgtttatttcgtgtgttatgtaacatatgtaatttctatctacttgcacaatgaaatatattcataaaataatcattttattgatttccaactgacttgaagtacactcggaaataaccatttacatatacatcggacttttgggaaggagcaccaacaatgtaaagggctccttcctttatatcaccaacatcacccgttgcactatttttccactctgtggacacacccaatctcttaaagaacttattacaggacttaacctgattacaaggcccaggcccataataagtagctccttgacctttggttgggtcaataccattgctctccaccacgcatgaccacgttttcttcaccacaaaacgatggcaaGAATTC

**Rb7 MAR**GAATTCtcgattaaaaatcccaattatatttggtctaatttagtttggtattgagtaaaacaaattcgaaccaaaccaaaatataaatatatagtttttatatatatgcctttaagactttttatagaattttctttaaaaaatatctagaaatatttgcgactcttctggcatgtaatatttcgttaaatatgaagtgctccatttttattaactttaaataattggttgtacgatcactttcttatcaagtgttactaaaatgcgtcaatctctttgttcttccatattcatatgtcaaaatctatcaaaattcttatatatctttttcgaatttgaagtgaaatttcgataatttaaaattaaatagaacatatcattatttaggtatcatattgatttttatacttaattactaaatttggttaactttgaaagtgtacatcaacgaaaaattagtcaaacgactaaaataaataaatatcatgtgttattaagaaaattctcctataagaatattttaatagatcatatgtttgtaaaaaaaattaatttttactaacacatatatttacttatcaaaaatttgacaaagtaagattaaaataatattcatctaacaaaaaaaaaaccagaaaatgctgaaaacccggcaaaaccgaaccaatccaaaccgatatagttggtttggtttgattttgatataaaccgaaccaactcggtccatttgcacccctaatcataatagctttaatatttcaagatattattaagttaacgttgtcaatatcctggaaattttgcaaaatgaatcaagcctatatggctgtaatatgaatttaaaagcagctcgatgtggtggtaatatgtaatttacttgattctaaaaaaatatcccaagtattaataatttctgctaggaagaaggttagctacgatttacagcaaagccagaatacaaagaaccataaagtgattgaagctcgaaatatacgaaggaacaaatatttttaaaaaaatacgcaatgacttggaacaaaagaaagtgatatattttttgttcttaaacaagcatcccctctaaagaatggcagttttcctttgcatgtaactattatgctcccttcgttacaaaaattttggactactattgggaacttcttctgaaaatagtg

**TM6 MAR**GAATTCaggtaatatttagaaatttaattaacataaccaaggatttttatatcggtaataactctaatatggtatccaaatcagtctagaactctcttacctctaataagtaaaagtacttctaataaattcatatactttttctctcttctccgatctctctttgctcttctttttatgtatcctttcctttctaatagccttttatgagaagtaaacttttagggttggccccccctccccccacaattatatagtttcttactcagttgttggaatataattcaaattcttaaataattgacggtgacattgagttttactttgtggaagagaattagattctcgtgttagtaaaatcggttagtaattgatgatgcattatttttactctataatagagatgcaattttatttttgcattttgggatcaaattgtaatgcagtcatatattgatttcataaatgtttgggatattgttggttatttaactagaaatagacttcttatttcatatttattgttaaaatcctttattggagatgaattatttgttcaacgattagaagttgatagtcgcttttgttttagaagaaattttaccgtagaccaagttaaggagttttagaagcactttgcatgggagcattagtgtatgttatggctttatcaaatataggttttgaagattcagagagccaagaaaagctagaacccaagaactaggaagttagagtaattcacaataccataacgtgatataaaactttttattgtaactcaaatcggtaatattttttgctttagtcttaatcgataaattatttttttatattgattagttataggaggctcaaaaagttgggaataattaaaatatcatattttgtatttgaacaatttatgaaatagtaattggtaaaaaatcactttaaatttttatcctatatccagaaggattatggtgtctggcatagttgtttggaagatttgaatcagggtaaaagtatgttgtaatttttattttgttataggcattttttgtgcttgattgttttgttgtcattatattttattatttggaagtgtatatatatgtttgattaaaatatagataatcaattttataagaaatttgcaacaattacaaaaggataaagtctacaatatgcgagtaaaatttgattgaacctaggatgtccctGAATTC

**Barley Yellow Dwarf Virus (BYDV) 5’ UTR**TCTCGAGtgaagattgaccatctcacaaaagctgttacgtgcttgtaacacactacacactcgttttgtattcgagaagtagttgcaacaacggtccccttattgcctgacaagctgagggccacccttctatccccaccgccacc

**Barley Yellow Dwarf Virus (BYDV) 3’ UTR**GGTACCagtgaagacaacaccactagcacaaatcggatcctgggaaacaggcagaacttcggttcataagctcgggtaggctgtcaacctaccgccgtatcgtattgtgtttggccgatggaggatcttcacgttatcgccgtttgtattcttgccttgactgtgctctctggggtaggcgctgttttgagttgctgccgttggtgctgcagcaatccttttcctccctccctctcttctgtttaagcaaaagactctcgatctgtgcgagagacaatcaaaaatatcgagggagcttcggctcagtgaggggattaacgacccccagtaatggccggtcctggcggacataaataacccgctataggacgaagtggtagccaccactgatcaaatggcaaacatgcttctgtgttgtacactgccccggagcctaccgggtcaacaaggctatcccaccaacccgatgaaatgagggtggagtgagcggagtgggtgacttcgtgatgtacacccgatcgtcaggattgaagacgttaaaactcgacgacctggtacaagtcgttaaactgactcgggtggatacaccacacccggcccagcatgttggcatacccacgatacgaaacgtgggtctcttggagccactacctgtgatgcaaggtagggtatgagtcttagcaagctctgagccaggagatggacataaaccatagcaatccaacgtgtaaccgcaatggggcaaacaacaggtgaaccgtgtccacgggcctggttaccgaaaggaaagccagtatccaacacagcaatgtgttgggggtcacaccctcggggtactcttaacgctgacactcgaaagagcagttcggcaacccGAGCTC

**Tumor Necrosis Virus-D (TNVD) 5’ UTR**

CTCGAGatacctaaccagtgtctcagtgattaagtaatcagctTCTAGAACA

**Tumor Necrosis Virus-D (TNVD) 3’ UTR**

GGTACCttgctttcatagatccgtcttcccagagacgttaagaagaagctggagaaaaatattaggttagaagcttgggcgtgacaaacccaagttgcatctcttacgtggttaatcacactgtatgttgacgtacaagccggatcctgggaaacaggtttaacggctcactgtggtggtgggccgtcgatacacttgtatgtgccccaatattggttgtcgagatctctctaggaacccGAGCTC

**Pea Enation Mosaic Virus (PEMV) 5’ UTR**

CTCGAgggtatttatagagatcagtatgaactgtgtcgctaggatcaagcggtggttcacacctgacttcacccctggcgagggcgtgaagtctacc

**Pea Enation Mosaic Virus (PEMV) 3’ UTR**

GGTACCagtgaagacaacaccactagcacaaatcggatcctgggaaacaggcagaacttcggttcataagctcgggtaggctgtcaacctaccgccgtatcgtattgtgtttggccgatggaggatcttcacgttatcgccgtttgtattcttgccttgactgtgctctctggggtaggcgctgttttgagttgctgccgttggtgctgcagcaatccttttcctccctccctctcttctgtttaagcaaaagactctcgatctgtgcgagagacaatcaaaaatatcgagggagcttcggctcagtgaggggattaacgacccccagtaatggccggtcctggcggacataaataacccgctataggacgaagtggtagccaccactgatcaaatggcaaacatgcttctgtgttgtacactgccccggagcctaccgggtcaacaaggctatcccaccaacccgatgaaatgagggtggagtgagcggagtgggtgacttcgtgatgtacacccgatcgtcaggattgaagacgttaaaactcgacgacctggtacaagtcgttaaactgactcgggtggatacaccacacccggcccagcatgttggcatacccacgatacgaaacgtgggtctcttggagccactacctgtgatgcaaggtagggtatgagtcttagcaagctctgagccaggagatggacataaaccatagcaatccaacgtgtaaccgcaatggggcaaacaacaggtgaaccgtgtccacgggcctggttaccgaaaggaaagccagtatccaacacagcaatgtgttgggggtcacaccctcggggtactcttaacgctgacactcgaaagagcagttcggcaacccGAGCTC

**Tobacco Mosaic Virus (TMV) 3’ UTR**

GGTACCggtagtcaagatgcataataaataacggattgtgtccgtaatcacacgtggtgcgtacgataacgcatagtgtttttccctccacttaaatcgaagggttgtgtcttggatcgcgcgggtcaaatgtatatggttcatatacatccgcaggcacgtaataaagcgaggggttcgaatccccccgttacccccggtaggggcccaGAGCT

**References**

Fernandez-Pozo, N., Menda, N., Edwards, J.D., Saha, S., Tecle, I.Y., Strickler,

S.R., Bombarely, A. et al. (2015) The Sol Genomics Network (SGN)–from

genotype to phenotype to breeding. Nucleic Acids Res. 43, D1036–D1041.
